# Role of endothelial‐to‐mesenchymal transition induced by TGF‐β1 in transplant kidney interstitial fibrosis

**DOI:** 10.1111/jcmm.13157

**Published:** 2017-04-04

**Authors:** Zijie Wang, Zhijian Han, Jun Tao, Jun Wang, Xuzhong Liu, Wanli Zhou, Zhen Xu, Chunchun Zhao, Zengjun Wang, Ruoyun Tan, Min Gu

**Affiliations:** ^1^ Department of Urology the First Affiliated Hospital Nanjing Medical University Nanjing China; ^2^ Department of Urology the Affiliated Nanjing Children's Hospital Nanjing Medical University Nanjing China; ^3^ Department of Urology Huai'an First People's Hospital Nanjing Medical University Huai'an China

**Keywords:** endothelial‐to‐mesenchymal transition, transforming growth factor‐beta1, kidney interstitial fibrosis, chronic allograft dysfunction, kidney transplantation, Smad, Akt/mTOR/p70S6K

## Abstract

Chronic allograft dysfunction (CAD) induced by kidney interstitial fibrosis is the main cause of allograft failure in kidney transplantation. Endothelial‐to‐mesenchymal transition (EndMT) may play an important role in kidney fibrosis. We, therefore, undertook this study to characterize the functions and potential mechanism of EndMT in transplant kidney interstitial fibrosis. Proteins and mRNAs associated with EndMT were examined in human umbilical vein endothelial cells (HUVECs) treated with transforming growth factor‐beta1 (TGF‐β1) at different doses or at different intervals with western blotting, qRT‐PCR and ELISA assays. Cell motility and migration were evaluated with motility and migration assays. The mechanism of EndMT induced by TGF‐β1 was determined by western blotting analysis of factors involved in various canonical and non‐canonical pathways. In addition, human kidney tissues from control and CAD group were also examined for these proteins by HE, Masson's trichrome, immunohistochemical, indirect immunofluorescence double staining and western blotting assays. TGF‐β1 significantly promoted the development of EndMT in a time‐dependent and dose‐dependent manner and promoted the motility and migration ability of HUVECs. The TGF‐β/Smad and Akt/mTOR/p70S6K signalling pathways were found to be associated with the pathogenesis of EndMT induced by TGF‐β1, which was also proven *in vivo* by the analysis of specimens from the control and CAD groups. EndMT may promote transplant kidney interstitial fibrosis by targetting the TGF‐β/Smad and Akt/mTOR/p70S6K signalling pathways, and hence, result in the development of CAD in kidney transplant recipients.

## Introduction

Renal transplantation (RT) is the optimal treatment for end‐stage renal disease 1, 2. However, the long‐term survival of allografts and recipients is limited to a decade on average because of the progressive deterioration of allograft function and CAD, which is a multifactorial condition associated with progressive kidney interstitial fibrosis 3, 4, 5, 6. Various immunological and non‐immunological risk factors are associated with kidney interstitial fibrosis, including acute and chronic rejection, oxidative stress, cytokine excess and calcineurin inhibitor toxicity 7, 8, 9, 10, 11. Current data confirm that epithelial‐mesenchymal transition (EMT), as a source of matrix‐generating myofibroblasts, is involved in the development of kidney interstitial fibrosis 12, 13. A common feature of fibrotic disorders is the presence of large numbers of activated myofibroblasts in affected tissues [14]. These cells play a crucial role in these fibrotic disorders by increasing the production of extracellular matrix (ECM), overexpressing α‐smooth muscle actin and reducing the expression of genes encoding ECM‐degradative enzymes 15, 16.

Recently, EndMT, which is considered as a distinctive type of EMT, was found to be an important source of activated myofibroblasts, which secrete and deposit excessive amounts of ECM in the kidney and thus contribute to the pathogenesis of renal fibrosis in chronic kidney diseases 17. EndMT is a complex biological process whereby endothelial cells lose their specific biomarkers, such as VE‐cadherin, von Willebrand factor (vWF), CD31 and CD34, and acquire a mesenchymal or myofibroblastic phenotype and consequently express mesenchymal cell markers, including α‐smooth muscle actin (α‐SMA), collagen I, collagen III and fibronectin (FN) 14. EndMT was first discovered in the process of cardiac development. Zeisberg showed that cardiac fibrosis is associated with the emergence of fibroblasts that originate from endothelial cells; this could mean that EndMT plays a crucial role in the pathogenesis of cardiac fibrosis 18. EndMT was also proven to be involved in cardiac fibrosis associated with acute viral myocarditis 19. In addition, EndMT was shown to play an important role in renal fibrosis. Zeisberg *et al*. reported that EndMT contributes to the accumulation of activated fibroblasts and myofibroblasts in renal interstitial fibrosis that occurs in chronic kidney diseases 20. Similarly, Li *et al*. confirmed that EndMT occurs and contributes to the generation of myofibroblasts in early diabetic renal fibrosis 21. EndMT is also involved in the development and progression of pulmonary fibrosis, hepatic fibrosis, intestinal fibrosis and wound healing, in addition to kidney fibrosis 14, 22. Based on these findings, we speculated that EndMT may contribute to the pathogenesis of renal interstitial fibrosis, which is one of the main factors associated with CAD in kidney transplant recipients. We believe that ours is the first such study to examine this potential role of EndMT in fibrosis associated with RT.

EndMT is controlled by a variety of biological mediators, such as growth factors, peptides and microRNAs 14, 23, 24. Among these factors, transforming growth factor‐beta (TGF‐β) is regarded as the most potent inducer of kidney fibrosis 25, 26. Therefore, in the present study, we aimed to clarify the role of TGF‐β1 in inducing EndMT during the development of kidney interstitial fibrosis associated with CAD in kidney transplant patients and elucidate the underlying mechanism. Our results showed that EndMT plays an important role in kidney interstitial fibrosis during the development of CAD through the TGF‐β/Smad and Akt/mTOR/p70S6K signalling pathways in kidney transplant recipients.

## Materials and methods

### Ethics statement

The study protocol was in accordance with the ethical standards of the Declaration of Helsinki and Istanbul. As the study was limited to the living‐related transplantation of kidney tissues to lineal or collateral relatives not beyond the third degree of kinship or cadaveric allograft donors of cardiac death, the protocol of this study was approved by the local ethics committee of the First Affiliated Hospital of Nanjing Medical University. Written informed consent was obtained from all transplant recipients and healthy volunteers. None of the transplant donors were from a vulnerable population, and all donors or next of kin freely provided their written informed consent.

### Reagents

Human recombinant TGF‐β1 was purchased from Santa Cruz Biotechnology (Santa Cruz, CA, USA). Antibodies against α‐SMA, VE‐cadherin, collagen I, collagen III, CD31, vWF and GAPDH were purchased from Abcam (Cambridge, MA, USA). Antibodies against phospho‐Smad 2 (Ser465/467), phosphor‐Smad 3 (Ser423/425), phospho‐Akt (Ser473), phospho‐mTOR (Ser2448), phospho‐p70S6K (Thr389), phospho‐Erk 1/2 (Thr202/Tyr204), phospho‐p38 MAPK (Thr180/Tyr182), phospho‐c‐Jun (Ser73), Smad 2, Smad 3, Akt, mTOR, p70S6K, Erk 1/2, p38 MAPK and c‐Jun, and selective inhibitors including SB431542, SB203580, UO126 and SP600125 were purchased from Cell Signaling Technology (Beverly, MA, USA). MK2206 was purchased from Selleckchem (Houston, TX, USA). Penicillin‐streptomycin and Roswell Park Memorial Institute (RPMI)‐1640 medium were purchased from Invitrogen (Life Technologies, Grand Island, NY, USA). Fetal bovine serum (FBS) was purchased from Gibco (Carlsbad, CA, USA).

### Sample collection

The samples included a total of 25 allograft segments, which were collected from transplanted kidney nephrectomy or kidney biopsy of recipients who underwent kidney transplantation between January 2001 and December 2010 at our centre and were diagnosed with CAD according to their clinical symptoms and allograft biopsy results. In addition, 25 normal kidney samples were collected through radical nephrectomy procedures, and each sample was obtained from more than 5 cm away from the tumour tissue. The baseline characteristics of patients in the CAD group and control group are given in Table 1.

**Table 1:** Baseline characteristics of the CAD and control groups

Clinical variables	CAD group	Control group	*P* value
*n*	25	25	
Age (years, mean ± S.D.)	32.68 ± 1.79	37.36 ± 2.23	NS
Male (%)	19 (76)	17 (68)	NS
BMI (kg/m^2^, mean ± S.D.)	23.89 ± 5.3	23.95 ± 5.0	NS
Transplant duration (years, range)	8.1 (6.5–10.2)		
Primary/secondary kidney transplant	25/0		
% PRA at transplant	0		
Donor source			
Living‐related	23		
Cadaveric	2		
Immunosuppressive regimen			
Prednisone + MMF + CsA	15		
Prednisone + MMF + Tac	10		
Biochemical parameters			
Serum creatinine (μmol/l, mean ± S.D.)	376.8 ± 18.60	91.80 ± 3.66	<0.0001
eGFR^a^ (min/1.73 m^2^, mean ± S.D.)	25.37 ± 2.53	77.33 ± 4.15	<0.0001

CAD: chronic allograft dysfunction, BMI: body mass index, PRA: panel reaction antibody, MMF: mycophenolate mofetil, CsA: cyclosporine A, Tac: tacrolimus, eGFR: estimated glomerular filtration rate, S.D.: standard deviation, NS: no significance.

a eGFR was estimated by the Cockcroft‐Gault formula: eGFR = (140 − age) × weight/72 × serum creatinine × (0.85 if female) 40.

### Cell extraction, identification and treatment

Primary HUVECs were extracted from umbilical cord segments (approximately 4 cm from an unclamped area of the cord). The vessels were rinsed free of blood with 10 ml M199/P/S/G and then inflated with M199/P/S/G/0.5% bovine serum albumin (BSA), which contained 2 mg/ml collagenase B, before they were clamped off for digestion. After the collagenase B solution and endothelial cells sheets from the inner surface were flushed with M199/P/S/G/0.5% BSA, the cells were pelleted by centrifugation (2 min. at 300 *g*). The cells that were recovered were transferred into 60‐mm dishes. The cells were continuously cultured on uncoated plastic dishes for two passages, and were then cultured for two to four passages for the *in vitro* experiments. All procedures in this experiment were in accordance with the institutional ethical guidelines.

HUVECs were cultured in RPMI‐1640 medium containing 10% FBS and 1% penicillin‐streptomycin in a humidified atmosphere containing 95% air and 5% CO_2_ at 37°C. Indirect immunofluorescence staining assay with the CD31 antibody was performed to identify the primary HUVECs extracted from the segments. The staining was visualized under a fluorescence microscope (Carl Zeiss, Oberkochen, Germany).

To evaluate the influence of TGF‐β1 on HUVECs, the cells were serum starved overnight and then treated with 0, 0.5, 1.0, 2.0, 5.0 and 10.0 ng/ml TGF‐β1 for 48 hrs or treated with 5 ng/ml TGF‐β1 for 0, 1, 6, 12, 24, 48 and 72 hrs. Total protein and RNA was extracted for western blot assays and quantitative real‐time PCR (qRT‐PCR), respectively.

To determine the mechanism underlying the effects of TGF‐β1, the cells were also treated with the TGF‐β1 receptor kinase (ALK5) antagonist SB431542, the Akt inhibitor MK2206, the p38 MPAK inhibitor SB203580, the Erk 1/2 inhibitor UO126 and the JNK inhibitor SP600125. HUVECs were serum starved overnight and pre‐treated with these selective inhibitors for 1 hr, and this was followed by stimulation with TGF‐β1 (5 ng/ml) for 48 hrs. The total protein of HUVECs was extracted for Western blot analysis. Every experiment described above was replicated at least three times.

### Quantitative real‐time PCR analysis

Total RNA was extracted from cells with the TRIzol reagent (Invitrogen). cDNA was synthesised with a PrimeScript™ RT reagent kit (TaKaRa Biotechnology, Shiga, Japan). qRT‐PCR was performed with a SYBR Green PCR kit (TaKaRa Biotechnology) on a DNA Engine Opticon 2 System (Bio‐Rad laboratories, Hercules, CA, USA). The specific primers used were as follows: ACTA2: (F) 5′‐AAAAGACAGCTACGTGGGTGA‐3′ and (R) 5′‐GCCATGTTCTATCGGGTACTTC‐3′, CDH5: (F) 5′‐TTGGAACCAGATGCACATTGAT‐3′ and (R) 5′‐TCTTGCGACTCACGCTTGAC‐3′, COL1A1: (F) 5′‐GAGGGCCAAGACGAAGACATC‐3′ and (R) 5′‐CAGATCACGTCATCGCACAAC‐3′, CD34: (F) 5′‐ACCAGAGCTATTCCCAAAAGACC‐3′ and (R) 5′‐TGCGGCGATTCATCAGGAAAT‐3′. mRNA expression was normalized to β‐actin expression. Every experiment described above was repeated at least three times.

### Western blot assay

The method used for the western blot assays have been described by Liu 27. Briefly, total proteins were extracted from cells or tissues, and the protein concentrations were determined with a BCA protein assay (Thermo Scientific, Waltham, MA, USA). Then, western blotting was performed by incubation of the cells with anti‐GAPDH (1:200), anti‐α‐SMA (1:2500), anti‐CD31 (1:1000), anti‐VE‐cadherin (1:1000), anti‐collagen I (1:5000), anti‐TGF‐β1 (1:250), anti‐Akt (1:1000), anti‐phospho‐Akt (1:1000), anti‐mTOR (1:1000), anti‐phospho‐mTOR (1:1000), anti‐p70S6K (1:1000), anti‐phospho‐p70S6K (1:1000), anti‐Erk 1/2 (1:1000), anti‐phospho‐Erk 1/2 (1:1000), anti‐p38 MAPK (1:1000), anti‐phospho‐p38 MAPK (1:1000), anti‐c‐Jun (1:1000) and anti‐phospho‐c‐Jun (1:1000) primary antibodies; this was followed by incubation with an anti‐rabbit or anti‐mouse secondary antibody (1:1000). The relative abundance of proteins was measured by using GAPDH expression as an internal reference, and the bands were quantified using an Odyssey infrared imaging system (LI‐COR biotechnology, Lincoln, NE, USA).

### Enzyme‐linked immunosorbent assay

The expression of collagen I and FN in the supernatant of HUVECs treated with the different concentrations of TGF‐β1 (0, 0.5, 1.0, 2.0, 5.0 and 10.0 ng/ml) for 48 hrs or treated with 5.0 ng/ml TGF‐β1 for various durations (1, 24, 48 and 72 hrs) was quantified using the PeliKine Compact human ELISA kit (BioLegend, San Diego, CA, USA) based on appropriate and validated sets of monoclonal antibodies. Assays were performed as described in the manufacturer's instructions. The assay was repeated at least three times independently.

### HUVEC motility assay

Motility assays with HUVECs were performed by plating cells in 6‐well culture dishes. The HUVECs were scratched with pipette tips and washed with phosphate‐buffered saline. Fresh RPMI‐1640 medium containing TGF‐β1 (5 ng/ml) was added to the scratched cells for 0, 24, 48 and 72 hrs. Images were taken using an inverted microscope (Eclipse TS100; Nikon, Shinagawa, Tokyo, Japan) at a ×100 magnification after incubation. Three random fields containing the migrated cells were selected and observed. The motility index was used to quantify the migrated cells by manual counting (motility index = the number of cells that migrated in the control group/the number of cells that migrated in the TGF‐β1 group). The assay was repeated at least three times independently.

### HUVEC migration assay

The effect of TGF‐β1 on the migration of HUVECs was examined by culturing the cells in 24‐well culture plates. HUVECs were seeded on the upper surface of polycarbonate filters with 8‐μm pores at a density of 5 × 10^4^ cells per upper chamber in a medium containing 5 ng/ml TGF‐β1. After 48 hrs of incubation at 37°C, non‐invasive cells on the upper chamber surface were removed by wiping with cotton swabs. Cell migration was quantified by counting the number of cells present on the lower surface using a phase‐contrast microscope (Eclipse TS100; Nikon) at a ×100 magnification. The migration index was used to quantify the migrated cells by manual counting (migration index = the number of cells that had migrated at 48 hrs /the number of cells that had migrated at 0 hr). The assay was repeated at least three times independently.

### Histological examination and immunohistochemical staining

Kidney tissue sections were stained with haematoxylin‐eosin (HE) for histopathological examination, and Masson's trichrome staining was used for quantification of collagen. The area that stained blue with Masson's trichrome was calculated by analysing 15 random fields per slide.

Immunohistochemical staining assays were performed to determine the distribution and degree of expression of TGF‐β1, α‐SMA, CD31 and collagen I/III in kidney tissues from kidney transplant recipients with CAD and the control group. The 5‐μm tissue sections that were obtained were deparaffinized in xylene and rehydrated in a graded series of alcohol. Non‐specific epitopes were blocked with 5% normal goat serum for 30 min. and then incubated overnight with anti‐collagen I (1:100), anti‐collagen III (1:100), anti‐TGF‐β1 (1:50), anti‐α‐SMA (1:200) and anti‐CD31 (1:50) primary antibodies at 4°C. Slices were incubated with biotinylated goat anti‐mouse/rabbit IgG (5.0 μg/ml; Abcam) for 1 hr. Images of immunohistochemically stained slides were captured, and quantitative analyses of positively stained cells were carried out independently by two authors with the help of a light microscope equipped with a digital camera (ECLIPSE 80i; Nikon).

### Indirect immunofluorescence double‐staining assay

Frozen tissue sections were blocked with goat serum for 30 min. at room temperature and then incubated with anti‐vWF (1:200) and anti‐α‐SMA (1:100) primary antibodies at 4°C overnight and with anti‐mouse/rabbit secondary antibodies (1:200; Santa Cruz Biotechnology) at 37°C for 2 hrs. vWF and α‐SMA exhibited red and green fluorescence, respectively, and the sections were observed with a laser scanning confocal microscope (Carl Zeiss LSM710).

### Statistical analysis

All data were expressed as the mean ± S.D. values determined from three independent experiments. The different treatment groups and control group were compared using two‐way analysis of variance (anova) followed by Dunnett's *posthoc* test. A *P* value less than 0.05 was considered to indicate statistical significance.

## Results

### Identification of HUVECs

**Figure 1:**
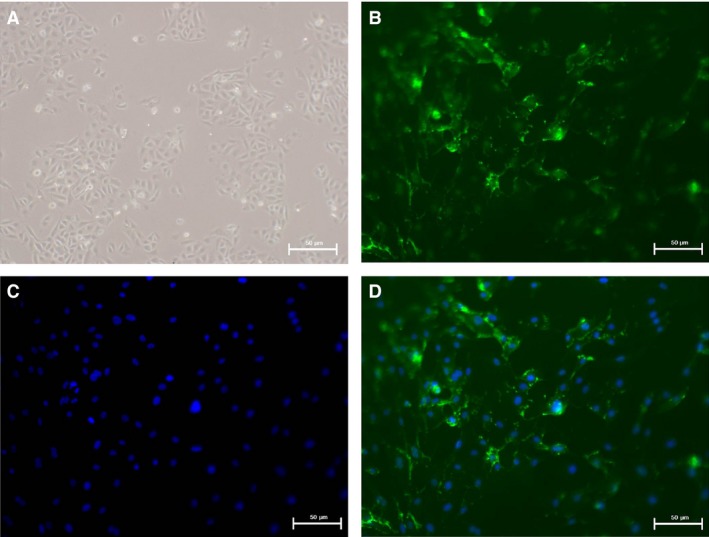
Indirect immunofluorescence staining of cells extracted from human umbilical cord segments. (**A**) The monolayer cells are observed to be arranged like ‘paving stones’ under the light microscope (×100). (**B**) CD31 (represented by green fluorescent signals) was mainly distributed in the cytoplasm of HUVECs (×200). (**C**) The nuclei of HUVECs were stained with DAPI (blue) (×200). (**D**) Positive staining signals for CD31 and DAPI (merged) in cells extracted from human umbilical cord segments prove that the cells were endothelial cells (×200).

In the early phase, the adherent HUVECs appeared small and triangular or spherical in shape and were found in a single cluster; further, the monolayer cells were observed to be arranged like ‘paving stones’ under the light microscope (Fig. 1A). Indirect immunofluorescence staining showed that the endothelial cell‐specific marker CD31 (represented by green fluorescent signals) was mainly distributed in the cytomembrane of HUVECs (Fig. 1B–D). Thus, we confirmed that the cells extracted from the umbilical cord segments were HUVECs.

### Effect of TGF‐β1 on the pathogenesis of EndMT and ECM secretion in HUVECs

**Figure 2:**
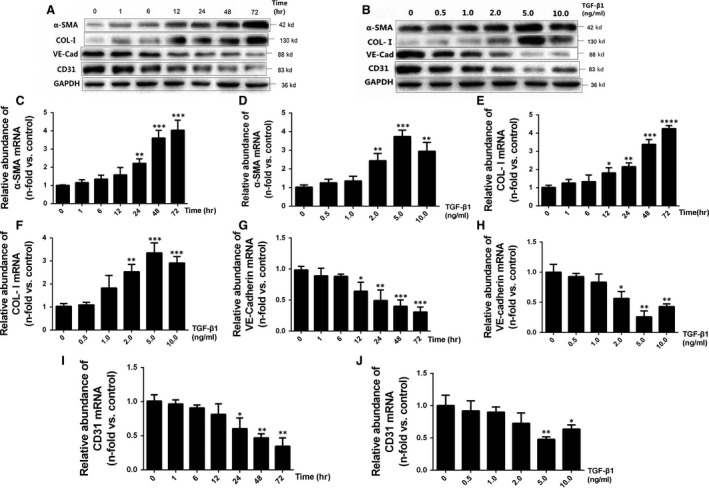
TGF‐β1 promotes α‐SMA and collagen I expression and suppresses VE‐cadherin and CD31 expression in the HUVECs. (**A** and **B**) Equal amounts of protein from whole cell lysates were analysed by western blotting with antibodies against α‐SMA, collagen I, VE‐cadherin, CD31 and GAPDH after incubation of HUVECs with 5 ng/ml TGF‐β1 for the indicated time points (**A**) or stimulation of HUVECs with various concentrations of TGF‐β1 for 48 hrs (**B**). As can be seen, TGF‐β1 promoted the expression of α‐SMA and collagen I and suppressed the expression of VE‐cadherin and CD31 in a time‐ and dose‐dependent manner. (**C–J**) HUVECs were stimulated with TGF‐β1 (5 ng/ml) for the indicated time points (C, E, G and I) or stimulated with various concentrations of TGF‐β1 for 48 hrs (**D, F, H** and **J**). Total RNA was isolated and reverse transcribed, and the resultant RNA was subjected to quantitative real‐time PCR to detect the gene expression of α‐SMA, collagen I, VE‐cadherin and CD31. The results of quantitative real‐time PCR were normalized to β2‐macroglobulin expression and expressed as the fold‐change relative to unstimulated control cells. Relative abundance of mRNAs is presented as the mean ± S.D. value of three independent experiments. The PCR results were in agreement with the western blot results. *P < 0.05, **P < 0.01, ***P < 0.001, ****P < 0.0001 *versus* the control group, as determined by one‐way anova (C‐J).

**Figure 3:**
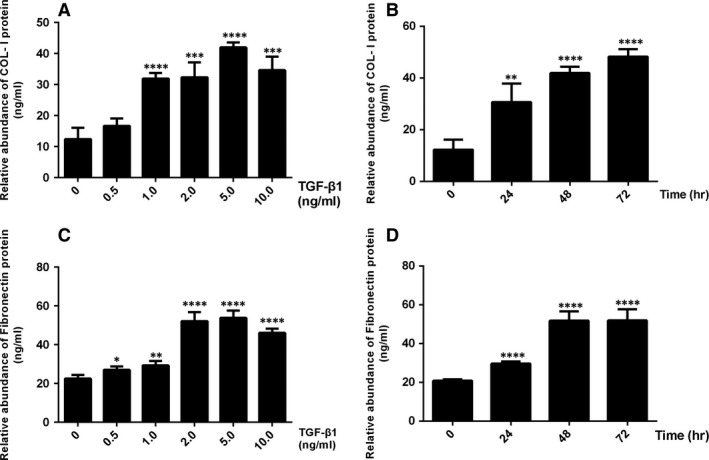
TGF‐β1 promotes extracellular matrix secretion in HUVECs. (**A–D**) HUVECs were incubated with various concentrations of TGF‐β1 for 48 hrs (**A** and **C**) or stimulated with 5 ng/ml TGF‐β1 for the indicated time points (**B** and **D**). The supernatant of the cultured HUVECs was collected for ELISA to determine the total concentration of collagen I (**A** and **B**) and fibronectin (**C** and **D**). The relative abundance of proteins was presented as the mean ± S.D. values of three independent experiments. *P < 0.05, **P < 0.01, ***P < 0.001, ****P < 0.0001 *versus* the control group by one‐way anova (**A**–**D**).

TGF‐β1 upregulated the protein and mRNA expression of α‐SMA and collagen I (Fig. 2A, C and E) and downregulated the protein and mRNA expression of VE‐cadherin and CD31 in a time‐dependent manner (Fig. 2A, G and I). This effect peaked at 72 hrs. Moreover, we observed that TGF‐β1 could induce dose‐dependent overexpression of α‐SMA and collagen I protein and mRNA (Fig. 2B, D and F), and lead to a dose‐dependent decline in the expression of VE‐cadherin and CD31 protein and mRNA (Fig. 2B, H and J) at doses of 0–5 ng/ml; however, these effects were reversed at concentrations of 10 ng/ml. Furthermore, a significant increase in the secretion of FN and collagen I in response to TGF‐β1 both in a concentration‐dependent and time‐dependent manner was detected (Fig. 3).

### Effect of TGF‐β1 on the motility and migration of HUVECs

**Figure 4:**
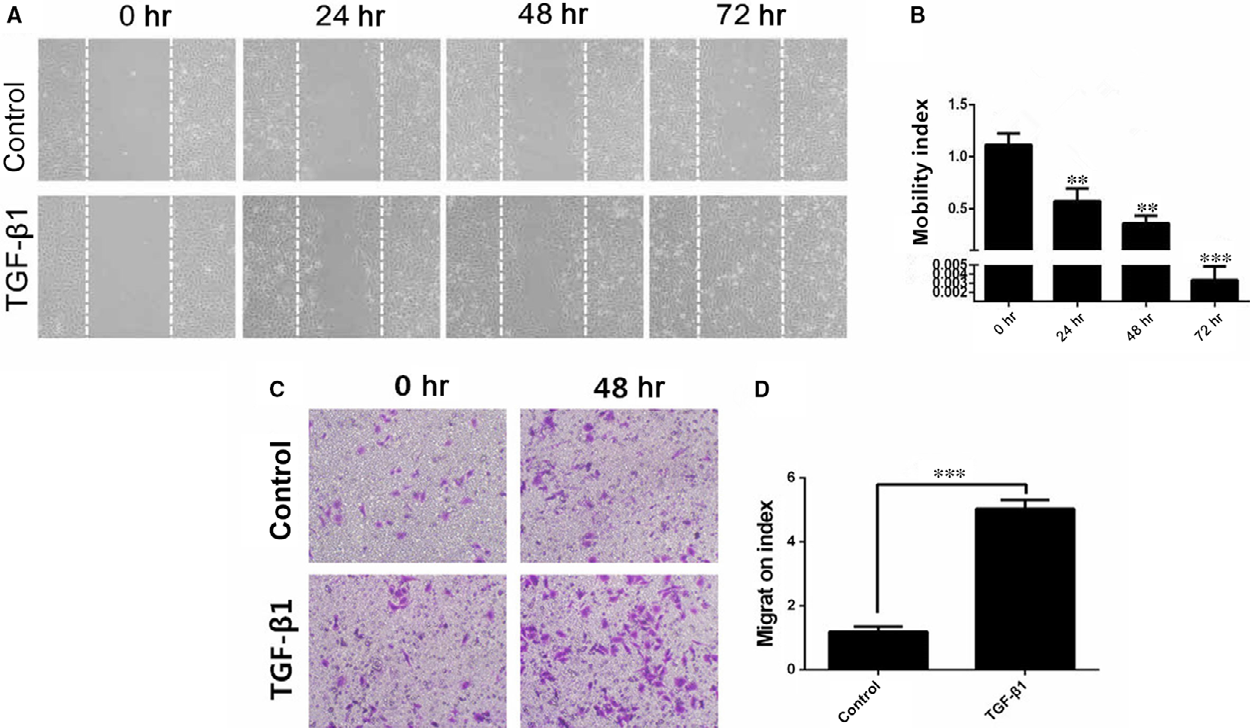
TGF‐β1 promotes the motility and migration of HUVECs. (**A** and **B**) HUVECs were wounded with a pipette and treated with 5 ng/ml TGF‐β1 for the indicated time points (**A**). The migrated cells were quantified by manual counting, and the motility index was determined using the formula ‘motility index = the number of cells that migrated in the control group/the number of cells that migrated in the TGF‐β1 group’ (**B**). (**C** and **D**) A total of 5 × 10^4^ HUVECs were seeded in the top chamber and treated with 5 ng/ml TGF‐β1 for the indicated time points (**C**). Cells that migrated through the membrane were stained and quantified. The migration index was determined using the formula ‘migration index = the number of cells that had migrated at 48 hrs/the number of cells that had migrated at 0 hr’ (**D**). The motility and migration indexes were expressed as the mean ± S.D. value of five independent experiments. **P < 0.01, ***P < 0.001 *versus* the control group, by one‐way anova (**B**) or Student *t* test (**D**).

TGF‐β1 significantly promoted the chemokinetic motility of HUVECs in a time‐dependent manner (Fig. 4A); this effect was the greatest at 72 hrs (Fig. 4B). Moreover, TGF‐β1 significantly promoted the chemotactic response and migration of HUVECs (Fig. 4C and D, *P* < 0.001 *versus* the control group).

### Effect of TGF‐β1 on the progression of EndMT *via* the TGF‐β/Smad and Akt/mTOR/p70S6K pathways in HUVECs

**Figure 5:**
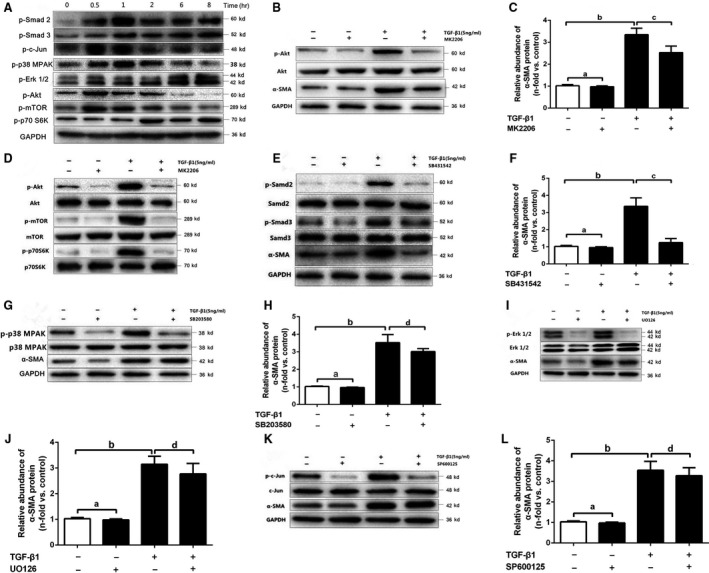
TGF‐β1 upregulates α‐SMA expression in HUVECs through the TGF‐β/Smad and Akt/mTOR/p70S6K signalling pathways. (**A**) HUVECs were treated with TGF‐β1 (5 ng/ml) for the indicated time points. Equal amounts of protein were collected from whole cell lysates and analysed by western blotting with antibodies against phosphorylated Smad 2, Smad 3, Akt, mTOR, p70 S6K, p38 MAPK, Erk1/2 and c‐Jun. (**B, D, E, G, I** and **K**) HUVECs were pre‐treated for 1 hr with MK2206 (10 μmol/l) (**B** and **D**), SB431542 (10 μmol/l) (**E**), SB203580 (10 μmol/l) (**G**), UO126 (10 μmol/l) (**J**) or SP600125 (5 μmol/l) (**K**), and specific chemical inhibitors of Akt, Smad, p38MAPK, Erk1/2 and JNK. Subsequently, cells were treated with TGF‐β1 (5 ng/ml) for the indicated time points. Cells were collected 1 hr after TGF‐β1 stimulation. Equal amounts of protein from whole cell lysates were analysed by western blotting with antibodies against phosphorylated and total Akt (**B** and **D**), phosphorylated and total mTOR (**D**), phosphorylated and total p70S6K (**D**), phosphorylated and total Smad 2 (**E**), phosphorylated and total Smad 3 (**E**), phosphorylated and total p38 MAPK (**G**), phosphorylated and total Erk1/2 (**I**), and phosphorylated and total c‐Jun (**K**). (**B, E, G, I** and **K**) HUVECs were collected 48 hrs after TGF‐β1 stimulation. Equal amounts of protein from whole cell lysates were analysed by western blotting with antibodies against α‐SMA and GAPDH. The ratio of α‐SMA to GAPDH density was expressed as the fold‐change relative to unstimulated control cells (C, F, H, J and L). The results of densitometric determination of the relative abundance of α‐SMA are presented as the mean ± S.D. value of three independent experiments. ^a^ *P* > 0.05 *versus* the control cells; ^b^ *P* < 0.01 *versus* the control cells; ^c^ *P* < 0.01 and ^d^ *P* > 0.05, cells treated with TGF‐β1 *versus* cells treated with TGF‐β1 and specific inhibitors, by Student *t* test (C, F, H, J and L).

To evaluate the signalling pathways involved in TGF‐β1‐induced EndMT in HUVECs, we examined phosphorylation of factors involved in the canonical and non‐canonical signalling pathways by western blotting. We observed activation of the Smad 2, Smad 3, Akt, mTOR, Erk 1/2, p38 MAPK and JNK signalling pathways in HUVECs treated with TGF‐β1 (Fig. 5A).

After HUVECs were pre‐treated with MK2206 (an Akt inhibitor), the expression of phosphorylated Akt and its downstream signalling molecules mTOR and p70S6K was significantly reduced after TGF‐β1 exposure for 1 hr (Fig. 5B and D). In a parallel set of experiments that were performed for 48 hrs, we found that pre‐treatment with MK2206 completely abolished the TGF‐β1‐mediated upregulation of α‐SMA (Fig. 5C). Similar results were observed in the experiments with the selective TGF‐βRI inhibitor SB431542 (Fig. 5E and F). In addition, we did not find any significant difference in α‐SMA protein expression in the experiments involving pre‐treatment with UO126 (an Erk1/2 inhibitor), SB203580 (a p38MAPK inhibitor) and SP600125 (a JNK inhibitor) for 1 hr (Fig. 5G–L); these findings indicate that these signalling pathways were not involved in the pathogenesis of TGF‐β1‐mediated EndMT.

### Effect of TGF‐β1 on EndMT through the TGF‐β/Smad and Akt/mTOR/p70S6K pathways in human kidney tissues from renal transplant recipients

**Figure 6:**
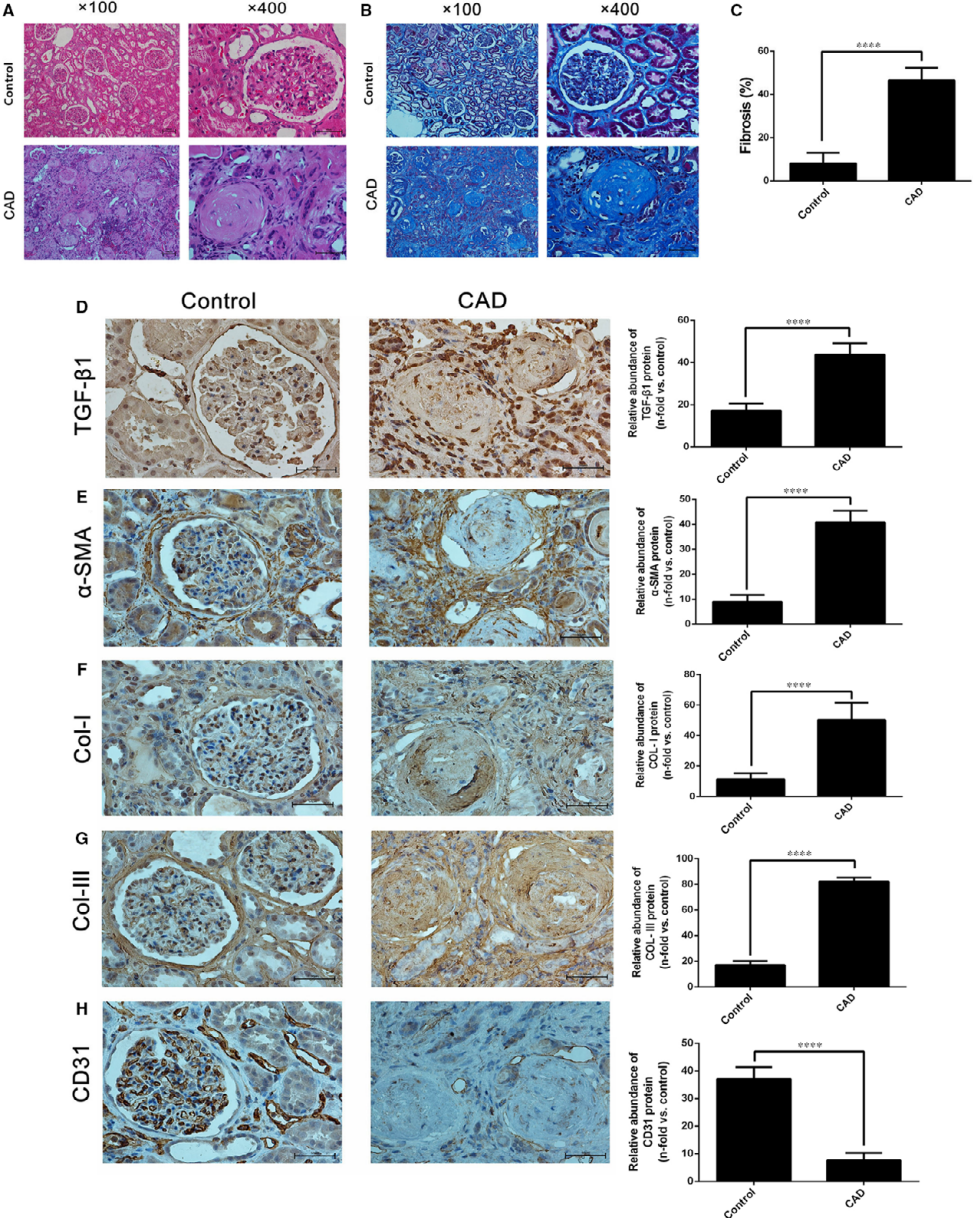
Increase in TGF‐β1, α‐SMA, collagen I and collagen III expression; decrease in CD31 expression; and kidney interstitial fibrosis in kidney tissues from renal transplant recipients with CAD. (**A** and **B**) Representative tissue sections from the control and CAD group were stained with HE (**A**) and Masson‐Trichrome (**B**) stain. (**C**) Quantitative analysis of the fibrosis intensity of kidney sections stained with Masson‐Trichrome was performed. The results demonstrated a significant degree of fibrosis in kidney tissues from renal transplant recipients with CAD. (**D–H**) Distribution and expression of TGF‐β1 (**D**), α‐SMA (**E**), collagen I (**F**), collagen III (**G**) and CD31 (**H**) in the CAD and control group were assessed by immunohistological staining assays. Fibrosis percentage and relative abundance of proteins were presented as the mean ± S.D. value of three independent experiments. Representative images of kidney tissues from the control group (*n* = 25) and CAD group (*n* = 25) are shown. ****P < 0.0001 *versus* the control group, as determined by the Student *t* test (**C**,** D**–**H**).

We performed a series of experiments with human specimens to confirm our *in vitro* findings. In the first set, histological studies with HE and Masson staining demonstrated significant renal interstitial fibrosis in kidney tissues from kidney transplant recipients with CAD compared with the control group patients (Fig. 6A–C). Moreover, the expression of TGF‐β1, α‐SMA, and collagen I/III was significantly higher and the expression of CD31 was remarkably lower in the CAD group than in the control group, according to the results of immunohistological staining (Fig. 6D–H).

**Figure 7:**
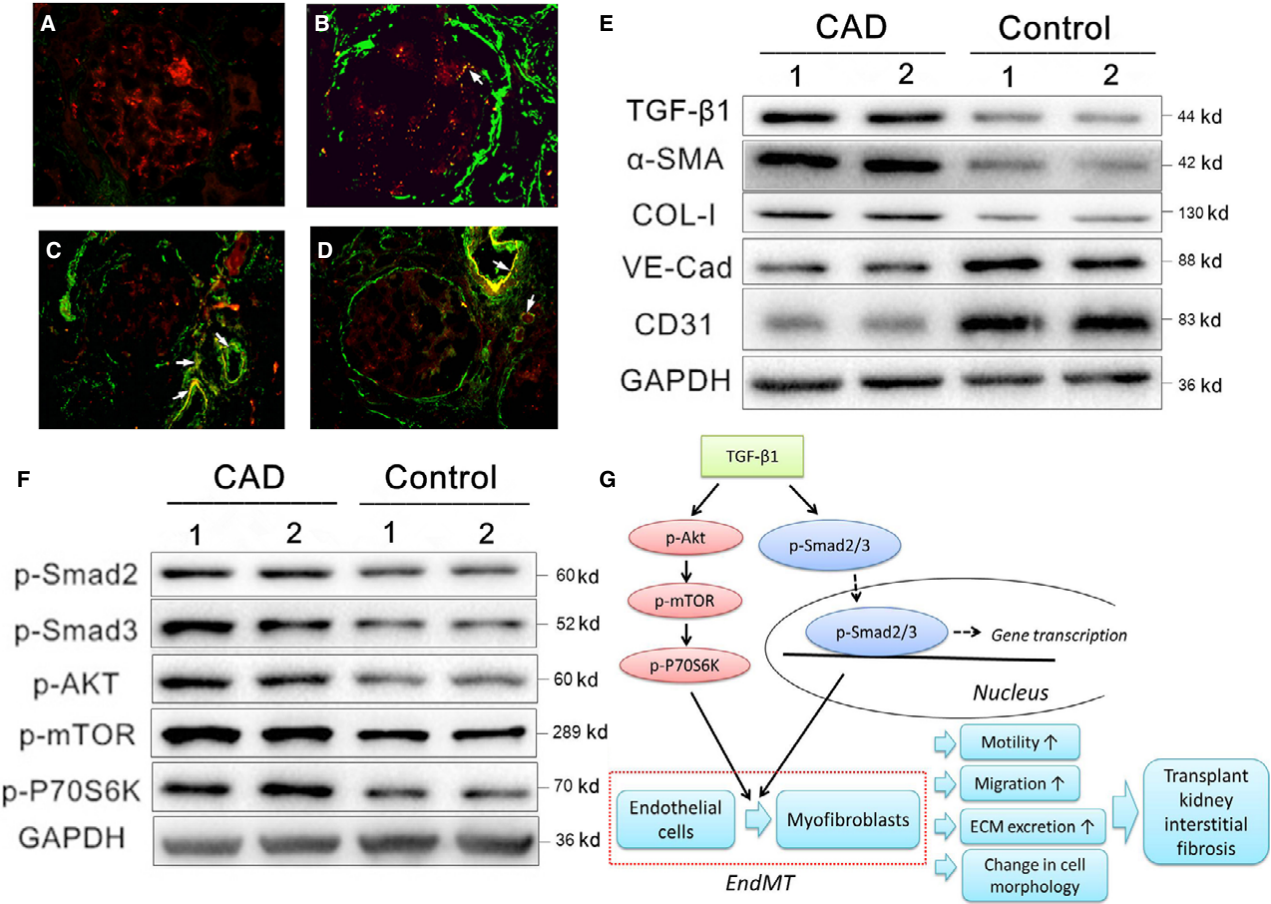
TGF‐β1 promotes the development of EndMT *via* the TGF‐β/Smad and Akt/mTOR/p70S6K signalling pathways in kidney tissues from renal transplant recipients with CAD. (**A–D**) Kidney tissues from the control group (**A**) and renal transplant recipients with CAD (**B–D**) were examined. The distribution and expression of α‐SMA (represented by green fluorescent signals) and vWF (represented by red fluorescent signals) were analysed by the indirect immunofluorescence double‐staining assay. (**E, F**) Equal amounts of proteins from human kidney tissues were analysed by western blotting with antibodies against TGF‐β1 (**E**), α‐SMA (**E**), collagen I (**E**), VE‐cadherin (**E**), CD31 (**E**), phosphorylated Smad 2 (**F**), Smad 3 (**F**), Akt (**F**), mTOR (**F**), p70 S6K (**F**) and GAPDH (**F**). (**G**) A model is proposed to illustrate the fibrotic mechanism involved in EndMT induced by TGF‐β1 in the pathogenesis of kidney interstitial fibrosis in kidney transplant recipients with CAD.

Indirect immunofluorescence double staining showed intensive positive staining for α‐SMA (represented by green fluorescence) (Fig. 7A), which was mainly distributed in the glomerular mesangium and vascular endothelial cells; double‐positive staining for vWF and α‐SMA (represented by yellow fluorescence) in the partial glomeruli and capillary endothelial cells; and relatively weak and discrete positive staining for vWF (represented by red fluorescence) in kidney tissues from the CAD group (Fig. 7B–D). Furthermore, western blot assays indicated upregulation of TGF‐β1, α‐SMA and collagen I and downregulation of VE‐cadherin and CD31 in the CAD group (Fig. 7E). Moreover, a significant increase in the phosphorylation activity of Smad 2, Smad 3, Akt, mTOR and p70S6K was found in the CAD group (Fig. 7F). Thus, these findings were in agreement with the *in vitro* results.

## Discussion

In the present study, we report that the progression of EndMT plays an important role in transplant renal interstitial fibrosis and CAD in kidney transplant recipients *via* the TGF‐β/Smad and Akt/mTOR/p70S6K signalling pathways (Fig. 7G). To the best of our knowledge, this is the first study to elucidate the role of EndMT and its underlying mechanism in transplant renal interstitial fibrosis and its relationship with CAD in kidney transplant recipients. Our results offer novel insights into the profound effects of EndMT and the mechanisms of transplant renal interstitial fibrosis, which may contribute to the treatment and prevention of CAD in kidney transplant recipients.

TGF‐β exerts its biological effects through both canonical Smad signalling pathways and non‐canonical pathways 28, 29. In the present study, we found that the TGF‐β/Smad and Akt/mTOR/p70S6K signalling pathways are required for TGF‐β1‐induced EndMT, whereas p38 MAPK, Erk 1/2 and JNK signals are not essential for this specific transduction process. Our results confirm those of a number of studies which have confirmed that TGF‐β1 is associated with the pathogenesis of EndMT 30, 31, 32. Both resident kidney cells and infiltrating leukocytes can secrete TGF‐β1, and canonical TGF‐β signal transduction involves consecutive processes that include binding of TGF‐β1 to TGF‐β receptor type II (TGF‐βRII), which recruits and phosphorylates TGF‐β receptor type I (TGF‐βRI) and subsequently activates Smad complexes, which consist of phosphorylated Smad 2/3 and the Co‐smad (Smad 4). These Smad complexes are then translocated into the nucleus, where they control gene transcription in conjunction with transcription factors 25, 29, 33. Various studies have reported that both Smad2 and Smad3, the two major downstream mediators of the TGF‐β/Smad signalling pathway, are highly activated in diabetic nephropathy, obstructive kidney disease and hypertensive nephropathy 34, 35, 36; similarly, our *in vivo* findings also show that these two proteins are activated in kidney transplant patients with CAD. Based on these previously published reports and our present findings, we propose that the TGF‐β/Smad signalling pathway is strongly associated with the molecular mechanism of TGF‐β1‐induced EndMT in transplant renal interstitial fibrosis.

Our *in vitro* findings indicate that the Akt/mTOR/p70S6K signalling pathway is involved in TGF‐β1‐induced EndMT. Akt, a known serine/threonine kinase, plays a central role in a range of cellular functions, including cell growth, proliferation, migration and protein synthesis 37. In addition, p70S6K, which is a downstream signalling molecule of Akt, regulates protein synthesis and proliferation, and could be blocked by its upstream kinase mTOR 38. Our results indicated the pivotal role of the Akt/mTOR/p70S6K signalling pathway in transplant renal interstitial fibrosis; it could therefore be a potential target for antifibrosis agents in the future. Our work also demonstrates that sirolimus, which is widely administered as a novel immunosuppressive agent in kidney transplantation, has antifibrotic effects in transplant kidneys because of its inhibitory effect on the mammalian target of mTOR 39.

In conclusion, the current study proves that the progression of EndMT could promote the pathogenesis of kidney interstitial fibrosis by targetting the TGF‐β/Smad and Akt/mTOR/p70S6K signalling pathways, and hence result in the development of CAD in kidney transplant recipients. The results of our study provide novel insight into the functions and mechanisms of EndMT, and prove that it is an additional target for the treatment and prevention of kidney interstitial fibrosis and CAD in kidney transplant recipients.

## Conflict of interest

The authors confirm that there are no conflicts of interest.
